# Associations between Coparenting Quality and Food Parenting Practices among Mothers and Fathers in the Guelph Family Health Study

**DOI:** 10.3390/nu13030750

**Published:** 2021-02-26

**Authors:** Sabrina Douglas, Gerarda Darlington, John Beaton, Kirsten Davison, Jess Haines, on behalf of the Guelph Family Health Study

**Affiliations:** 1 Department of Family Relations and Applied Nutrition, University of Guelph, Guelph, ON N1G2W1, Canada; beaton@uoguelph.ca (J.B.); jhaines@uoguelph.ca (J.H.); 2 Department of Mathematics and Statistics, University of Guelph, Guelph, ON N1G2W1, Canada; gdarling@uoguelph.ca; 3 Boston College School of Social Work, Chestnut Hill, MA 02467, USA; kirsten.davison@bc.edu

**Keywords:** coparenting, food parenting, parenting practices, family, nutrition

## Abstract

Coparenting quality and food parenting practices have been shown to have a strong influence on child outcomes. However, little is known about whether coparenting quality may influence food parenting practices. This study aimed to investigate how coparenting quality is associated with both mothers’ and fathers’ food parenting practices. A cross-sectional analysis was conducted of 58 mothers and 40 fathers enrolled in the Guelph Family Health Study. The Coparenting Relationship Scale and the Comprehensive Feeding Practices Questionnaire were used to measure coparenting and food parenting practices, respectively. Linear regressions using generalized estimating equations were used to examine associations between coparenting quality and food parenting practices in mothers and fathers. Among mothers, higher coparenting quality was associated with lower use of food for emotional regulation, restriction of food for health, and child control of food intake and with higher encouragement of a balanced and varied diet, provision of a healthy home environment, and modeling of healthy eating behaviors. Among fathers, higher coparenting quality was associated with lower pressure to eat and with higher encouragement of a balanced and varied diet and provision of a healthy home environment. Coparenting quality is associated with food parenting practices among both mothers and fathers. Interventions aiming to improve food parenting practices should include fathers and should consider targeting parents’ coparenting relationship.

## 1. Introduction

Diet and nutrition are vital for the growth and development of toddler and preschool-aged children. Poor dietary intakes may lead to an increased risk of adverse health outcomes, such as diabetes, cardiovascular disease and cancer [1]. Parents act as food gatekeepers to young children. Therefore, their food parenting practices are instrumental in determining the eating behavior and nutritional status of their children. Food parenting practices have been defined as the actions taken by parents to influence their children’s eating [2]. Certain food parenting practices have been associated with better health outcomes and behaviors among children, whereas others have been associated with more adverse health outcomes and behaviors. Restriction of food, which refers to parents controlling a child’s access to certain foods, has been shown to be associated with preoccupations with foods and higher body mass index in children [3,4,5]. Pressuring to eat refers to when parents insist or demand children consume certain foods and have been shown to be predictive of child food fussiness [6]. Using food as a reward for behavior and using food to control children’s emotions are food parenting practices that have been associated with higher energy-dense snacking and higher body mass index [7,8]. Food parenting practices that have been associated with better eating and weight outcomes in children include parents monitoring children’s food intake, modeling healthy eating behaviors, allowing children to control what and how much they eat, providing a healthy home food environment, and encouraging balance and variety of foods [9,10,11,12]. While there is substantial research showing that food parenting practices can impact children’s eating behavior and weight outcomes, most of the research has only considered mothers’ food parenting practices, with less research examining the role of fathers’ food parenting practices [13,14].

Coparenting quality, which refers to the ways that parents do or do not coordinate with and support each other in their roles as parents, has been shown to be associated with both child and parent outcomes and wellbeing. Coparenting quality is multi-determined by many factors, including couple relationship quality, parent negative mood, parenting stress, and work hours [15]. Coparenting undermining has been shown to be associated with poorer parental wellbeing and efficiency, while coparenting support has been associated with better parental wellbeing and efficiency [16]. These results indicate that parents who agree more about how they parent children together may be more comfortable raising children and feel like they are supported in the process [16]. Importantly, research shows that there are no gender differences in how coparenting was associated with parental wellbeing and efficiency, indicating that coparenting is relevant for parental adjustment among both mothers and fathers [16]. One Canadian study observed a positive association between unsupportive coparenting and child maladjustment in school-age children [17], and research has shown that coparenting can be more predictive of child outcomes, such as attachment security and behavior problems, than is general marital quality [18,19]. Considering the strong association between coparenting and child and parent outcomes and the fact that feeding children is a regular household routine that likely requires coordination and support across caregivers, coparenting quality may also influence food parenting practices.

To our knowledge, there have been no quantitative studies that have examined the association between coparenting quality and food parenting practices. Gevers and colleagues measured the moderation effect of parental feeding incongruence on the association between food parenting practices and adolescent snacking and found that high levels of parental disagreement with regards to feeding attenuated the association between restrictive food parenting practices of fathers and adolescents’ snacking behavior [20]. These results indicate that restrictive practices may limit snacking behavior in adolescents, but only if parents’ food parenting practices are in agreement. A few qualitative studies have explored the role of coparenting in child feeding [21,22,23]. One qualitative study of fathers explored cooperative and conflicting food parenting practices between parents and found that fathers reported that conflicting food parenting practices between mothers and fathers often resulted in child tantrums or refusal to eat [21]. A second qualitative study found that, while mothers and fathers tended to agree that family mealtimes and healthy meals are important, they reported disagreement in the strategies used to limit certain foods [22]. A third qualitative study with parents of children six months to three years revealed that fathers reported that coparenting quality influenced their food parenting practices throughout their children’s early life [23].

Results from these existing studies suggest that coparenting quality may be an important but understudied aspect of the family feeding environment. This study builds on this existing literature by investigating how coparenting quality is associated with mothers’ and fathers’ food parenting practices. Understanding how coparenting quality may influence food parenting practices will help inform the development of family-based interventions designed to promote positive food parenting practices that support healthful eating behavior among young children.

## 2. Materials and Methods

### 2.1. Recruitment and Eligibility

Data were obtained from phase 1 and phase 2 of the Guelph Family Health Study (GFHS) pilots between December 2014 and August 2016. The GFHS is a randomized control trial of a home-based obesity prevention intervention in Guelph, Ontario [24]. The intervention provided in phase 1 has been described previously [24]. Phase 1 and phase 2 received similar interventions, which did not directly address coparenting quality or food parenting practices. Families were eligible to participate if they had at least one child between 2.5 and 7 years old if they lived in Guelph or Wellington County, and they were not planning on moving in the next year. A total of 147 parents from 86 families were recruited at baseline. The present study used 2-year follow-up data from phase 1 and 1-year follow-up data from phase 2 as these were the survey timepoints when coparenting quality was assessed. Of the parents, who participated in the baseline, 49 participants were excluded from these analyses for the following reasons: Not living with a partner or spouse (*n* = 6) so were not asked the coparenting questions, missing covariates (*n* = 5), missing components of the coparenting or food parenting practices survey (*n* = 2) and no survey response (*n* = 36). The final analytic sample consisted of 58 mothers and 40 fathers from 61 families. Parents contributed a total of 141 observations since some families had more than one child enrolled. The study was approved by the University of Guelph Research Ethics Board (REB14AP009), and all participants provided written informed consent.

### 2.2. Food Parenting Practices Measure

To assess food parenting practices, mothers and fathers answered questions from the Comprehensive Feeding Practices Questionnaire (CFPQ) [25]. The CFPQ is a 42-item questionnaire that assesses 12 different food parenting practices. Items assessing two practices, “restriction for weight control” and “teaching about nutrition,” were excluded from the GFHS survey as those items overlapped with other items included in the survey. One of the 5 items in the “child control” section was also excluded because of an error in the question. The GFHS survey assessed 10 food parenting practices, which can be divided and described in terms of Vaughn et al.’s categories of coercive control, structure and autonomy support practices [26]. Coercive control practices are centered around serving the goals of the parent without taking the child’s needs into consideration. The coercive control practices measured in this study included: using food to regulate child emotions (3 items), using food as a reward (3 items), pressuring to eat (4 items), and restricting food for health (4 items). Structure refers to how parents organize the food environment for their children using noncoercive practices and are measured from practices, including providing a healthy home environment (4 items), modeling healthy eating behaviors (4 items), monitoring unhealthy foods (4 items), and allowing the child to control his/her food intake (unstructured practice) (4 items). Autonomy support refers to how parents promote independence for their children and are measured from practices, including involving children in meal planning and preparation (3 items) and encouraging balance and variety (4 items). Items were rated on a 4-point Likert scale (strongly disagree to strongly agree). Responses were coded into a numerical score from 1 to 4 and then averaged to create a score for each food parenting practice. Higher scores indicate more agreement with the food parenting practice. The CFPQ was completed by parents for each child enrolled in the study. Therefore, parents with more than one child in the GFHS contributed more than one observation toward the overall score for each food parenting practice. The internal consistency (Cronbach’s alpha) for all CFPQ subscales were > 0.7 among mothers and fathers in our sample (data not shown), except for mothers’ involving children in meal planning and prepping (α = 0.67), and mothers’ allowance of the child to control food intake/unstructured practices (α = 0.53).

### 2.3. Coparenting Quality Measure

To assess coparenting, mothers and fathers completed the coparenting relationship scale (CRS) brief version [27], which includes 14 items that assess 7 different aspects of coparenting quality, including coparenting agreement, coparenting closeness, exposure to conflict, coparenting support, coparenting undermining, endorse partner parenting and division of labor. Items were rated on a 6-point Likert scale. A total score was created by averaging the responses to all the items on the brief version of the survey with a higher score indicating more positive coparenting quality.

### 2.4. Statistical Analysis

Statistical analyses were performed using SAS University Edition version 3.8 [28]. To account for possible correlation among outcomes within families, linear regressions using generalized estimating equations were used to determine associations between the independent variable, coparenting quality, and each of the dependent variables: child control, emotional regulation, encouragement of balance and variety, environment, food as reward, involvement, modeling, monitoring, pressure, and restriction for health. All analyses were stratified by parent gender. Total family income (reported by the first parent in a family to enroll in the study), parental ethnicity, child age, and intervention status were included as covariates. Intervention status was not an effect modifier for any model. Therefore, families were not stratified by intervention status in the analyses. Statistical significance was determined as *p* < 0.05.

## 3. Results

### 3.1. Sample

Participant characteristics are presented in Table 1. The mean age of parents was 38.7 (4.6) years, 59% were female, and 63% of families had a yearly household income over $100,000. Approximately 88% of participants identified as being Caucasian. Mothers’ and fathers’ coparenting quality and food parenting practices scores are shown in Table 2.

### 3.2. Associations between Coparenting Quality and Food Parenting Practices among Mothers and Fathers

Coparenting quality among mothers was inversely associated with use of food for emotional regulation (β = −0.20; 95% confidence interval (CI), −0.34 to −0.07; *p* = 0.003), restricting food for health (β = −0.19; 95% CI, −0.34 to −0.03; *p* = 0.02), and allowing their child to control food intake/unstructured practices (β = −0.14; 95%, −0.25 to −0.04; *p* = 0.01). Coparenting quality among mothers was positively associated with encouragement of a balanced and varied diet (β = 0.13; 95% CI, 0.04 to 0.23; *p* = 0.01), provision of a healthy home environment (β = 0.26; 95% CI, 0.17 to 0.35; *p* < 0.0001), and modeling of healthy eating behaviors (β = 0.12; 95% CI, 0.01 to 0.23; *p* = 0.04). Coparenting quality was not significantly associated with maternal use of pressure to eat, use of food as a reward, monitoring intake of unhealthy foods, and involvement of children in meal planning and preparation (Table 3).

Coparenting quality among fathers was inversely associated with pressure to eat (β = −0.19; 95% CI, −0.34 to −0.05; *p* = 0.01; Table 3), and positively associated with encouragement of a balanced and varied diet (β = 0.11; 95% CI, −0.004 to 0.23; *p* = 0.04), and provision of a healthy home environment (β = 0.24 95% CI, 0.10 to 0.39; *p* = 0.001). Coparenting quality was not significantly associated with paternal use of restriction for health, use of food for emotional regulation, using food as a reward, monitoring intake of unhealthy foods, modeling of healthy eating behaviors, allowing their child to control food intake/unstructured practices, and involvement of children in meal planning and preparation (Table 3).

## 4. Discussion

To our knowledge, this is the first study to examine quantitative associations between coparenting quality and food parenting practices. In this sample of Canadian families with preschool children, mothers’ and fathers’ self-reported coparenting quality was associated with food parenting practices. Among mothers, higher coparenting quality was associated with lower use of food for emotional regulation, restriction of food for health, and allowing the child to control food intake and associated with higher encouragement of a balanced and varied diet, provision of a healthy home environment, and modeling of healthy eating behaviors. Among fathers, higher coparenting quality was associated with lower pressure to eat and associated with higher encouragement of a balanced and varied diet and provision of a healthy home environment. These results suggest that coparenting quality in both mothers and fathers is an important aspect of the home feeding environment, independent of household income, parent ethnicity, and intervention status.

Overall, our results show that higher coparenting quality is associated with more positive food parenting practices. This is consistent with previous literature showing that higher coparenting quality is associated with better child outcomes and general parenting outcomes [16,29,30]. Previous studies have found that higher coparenting quality among parents is associated with better psychological adjustment and fewer behavior problems among children [29,30]. Additionally, among mothers and fathers, coparenting support has been associated with lower stress and higher parenting efficiency [16]. Taken together, this previous research, as well as our results, suggests that coparenting quality is an important predictor of both parent and child outcomes that should be considered within family-based research.

While some research has found that food parenting practices differ somewhat between mothers and fathers [22,31,32], limited research has explored potential reasons for these gender differences in food parenting practices. This study provides modest evidence that coparenting might play a role in these differences. Our study found more significant associations between coparenting quality and food parenting practices among mothers than among fathers. These findings could be explained by previous research findings that mothers are more involved with feeding children than fathers [22,33], and therefore could suggest that coparenting quality is more meaningful to mothers in the context of child feeding. Results showed that, while coparenting quality was significantly associated with the encouragement of a balanced and varied diet and provision of a healthy food environment among both mothers and fathers, significant associations between coparenting and use of food for emotional regulation, modeling of healthy eating behaviors, restriction for health and allowance of children to control food intake/unstructured practices was only found among mothers. Thus, our results suggest that coparenting quality may be more relevant for mothers with regards to their food parenting than for fathers.

Results from this study also highlight that coparenting quality may be more relevant for some food parenting practices. For example, more consistent findings were found between coparenting quality and structure food parenting practices than coercive control or autonomy support food parenting practices. Structure and autonomy support practices have been found to be associated with more healthful eating behaviors among children [9,10,11], while coercive control practices have been shown to be associated with less healthful eating behavior and higher weight outcomes among children [3,4,5,7,8,12]. Structure food parenting practices refer to practices that help to organize a child’s food environment, including providing a healthy home environment, meal and snack routines, and monitoring the intake of unhealthy foods. One potential explanation for why coparenting quality may be more strongly associated with structured food parenting practices could be that these practices require a more organized environment as well as more of a shared approach to feeding. Coparenting quality scores include measures on joint family management, support, childrearing agreement, and division of labor. Higher scores in these aspects of coparenting may allow for a more shared approach to feeding, which may be particularly important to the structure-based food parenting practices. For example, creating a regular meal routine or ensuring a healthy home food environment likely requires more collaboration and support from both parents than some of the more individual-focused food parenting practices, such as pressuring a child to eat or encouraging healthy eating. These results are supported by qualitative research by Tan and colleagues, which found that couples reported that routines around feeding and mealtime (a structured food parenting practice) are important in order to jointly manage feeding responsibilities [22].

There are some limitations of this study that need to be considered. No causal links can be made between coparenting quality and food parenting practices due to the cross-sectional design of the study. The sample consisted mostly of families with middle to high socioeconomic status and white-identifying parents; thus, the results may not be generalizable to lower-income families or those from other races and ethnic groups. The sample was limited to parents who reside with a coparent. This may have biased our sample toward families with parents who have better communication and coparenting quality. The coparenting relationship scale and the Comprehensive Feeding Practices Questionnaire were measured by self-report, which increases the risk for social desirability bias and measurement error, which could bias our results towards the null. Additionally, we only included the overall coparenting quality score and not the individual scores, such as division of labor and undermining, as part of the analysis. Future research could examine the components of coparenting in relation to food parenting practices. The Coparenting Relationship Scale is a general measure of coparenting and is not designed to measure the quality of coparenting, specifically regarding feeding. Future research should consider measuring coparenting constructs specific to the feeding context [32]. In addition, future research should consider including the measurement of child eating behaviors and the potential moderating effect of coparenting on the relationship between food parenting practices and child eating behavior.

Overall, we found that higher coparenting quality was associated with more positive food parenting practices among both mothers and fathers; however, more consistent results were found among mothers and for structure-based food parenting practices. Given that parents act as food gatekeepers to children and that food parenting practices impact children’s food intake and nutrition, these findings suggest that coparenting quality is an important consideration in child feeding research. Future research should consider measuring coparenting quality specific to the feeding context and should include a more diverse sample of families to allow for more generalizable results.

## Figures and Tables

**Table 1:** Characteristics of mothers and fathers in the Guelph family health study pilot.

Variable	Parents (*n* = 98)	Mothers (*n* = 58)	Fathers (*n* = 40)
Sex, %			
Female	59.2		
Male	40.8		
Age, y; mean (SD)	38.7 (4.6)	38.0 (4.1)	39.8 (5.0)
Household income *, %			
<$40,000	6.6		
$40,000–$59,999	4.9		
$60,000–$79,999	16.4		
$80,000–$99,999	9.8		
$100,000–$149,999	36.1		
>$150,000	26.2		
Ethnicity, %			
Caucasian	87.8	86.2	90.0
Other	12.2	13.8	10.0

* family level variable.

**Table 2:** Mean scores on the comprehensive food parenting practices questionnaire and the coparenting quality questionnaire among mothers and fathers.

Food Parenting Practice	Mothers’ Mean Score (SD), *n* = 58	Fathers’ Mean Score (SD), *n* = 40
Restriction for health	2.70 (0.69)	2.61 (0.56)
Pressure to eat	2.30 (0.61)	2.35 (0.48)
Use of food for emotion regulation	1.57 (0.57)	1.53 (0.54)
Use of food as a reward	1.97 (0.65)	2.10 (0.74)
Monitoring intake of unhealthy foods	3.16 (0.63)	3.26 (0.67)
Modeling healthy eating behaviors	3.33 (0.48)	3.26 (0.53)
Allowing child to control food intake	2.17 (0.44)	2.07 (0.54)
Provides a healthy home environment	3.27 (0.47)	3.23 (0.51)
Involving children in meal planning and prepping	2.84 (0.48)	2.75 (0.47)
Encouragement of a balanced and varied diet	3.48 (0.43)	3.52 (0.44)
Coparenting quality	4.67 (1.03)	4.91 (0.83)

**Table 3:** Results of linear regressions using generalized estimating equations of mother and father coparenting quality scores with food parenting practices scores.

Food Parenting Practice	Mothers		Fathers	
	Adjusted Estimate * (95% CI)	P	Adjusted Estimate * (95% CI)	P
Restriction for health	−0.19 (−0.34, −0.03)	0.02	−0.20 (−0.42, 0.01)	0.06
Pressure to eat	−0.11 (−0.28, 0.05)	0.16	−0.19 (−0.34, −0.05)	0.01
Use of food for emotion regulation	−0.20 (−0.34, −0.07)	0.003	−0.14 (−0.33, 0.05)	0.14
Use of food as reward	−0.02 (−0.18, 0.15)	0.85	−0.04 (−0.32, 0.25)	0.80
Monitoring intake of unhealthy foods	0.10 (−0.09, 0.30)	0.31	−0.16 (−0.46, 0.14)	0.31
Modeling healthy eating behaviors	0.12 (0.01, 0.23)	0.04	0.10 (−0.08, 0.27)	0.28
Allowing child to control food intake	−0.14 (−0.25, −0.04)	0.01	0.06 (−0.15, 0.26)	0.58
Provides a healthy home environment	0.26 (0.17, 0.35)	<0.0001	0.24 (0.10, 0.39)	0.001
Involving children in meal planning and prepping	0.10 (−0.02, 0.22)	0.11	0.03 (−0.10, 0.16)	0.61
Encouragement of a balanced and varied diet	0.13 (0.04, 0.23)	0.01	0.11 (0.004, 0.23)	0.04

* model was adjusted for household income, parent ethnicity, child age, and intervention status.

## Data Availability

The Guelph Family Health Study (GFHS) welcomes outside collaborators. Interested investigators can contact GFHS investigators to explore this option, which preserves participant confidentiality and meets the requirements of our Research Ethics Board, to protect human subjects. Due to Research Ethics Board restrictions and participant confidentiality, we do not make participant data publicly available.
